# Role of leadership and incentive-based programs in addressing vaccine hesitancy in India

**DOI:** 10.1016/j.jvacx.2023.100346

**Published:** 2023-07-07

**Authors:** Bita Afsharinia, Anjula Gurtoo

**Affiliations:** Senior Research Scholar, Department of Management Studies, Indian Institute of Science, Bangalore 560012, India

**Keywords:** COVID-19, Vaccine hesitancy, India, Leadership, Incentives, Social norms

## Abstract

The current study goes beyond the popular 5Cs model of vaccine hesitancy to explore perceived social norms, role of leadership and the role of incentive-based intervention for improving the likelihood of vaccination, in a population-representative sample of adults in India (N = 1129). We investigated differences in socio-demographic status, perceived descriptive norms like vaccine behaviours, beliefs, experience and perceived injunctive norms like community and political leadership within the context of the COVID-19 pandemic are related to vaccination. Further, we examined the role of incentive-based intervention as a potentially salient predictor of adults’ vaccine uptake. The study is based on a cross-sectional survey conducted in July-November 2021, which included questions about impacts of pandemic collected by phone call interview from adults’ beneficiaries of Government free food schemes, across 11 states in India. The data include 81 % vaccinated and 19 % vaccine hesitant participants, with a higher proportion of business or self-employed (53.1 %) as vaccine hesitant. We performed the *t*-test to assess the variation between vaccinated and vaccine hesitant adults, and Exploratory Factor Analysis to uncover the underlying dimension of vaccine hesitancy. Subsequently, Binary Logistic Regression model probed the factors associated with likelihood of vaccination uptake. In contrast to the prevailing evidence, results demonstrated community and political leadership has significant negative impact on vaccination (p-value < 0.06, 95 % CI, 0.57–1.01). Incentive-based intervention offer a vital leverage to enhance positive attitude towards vaccination (p-value < 0.05, 95 % CI, 1.01–1.80). The results thus broadly highlight the limited role of political and community leaders in leveraging their influence on vaccination, the role of timely, accurate information, applications of telemedicine as an important tool to provide healthcare and the need for optimal design of incentive-based vaccination programs recommended by trusted sources.

## Highlights

• Study goes beyond the popular vaccine hesitancy 5Cs model to explore social norms.
• Perceived descriptive norms and perceived injunctive norms are explored.
• Limited role of political and community leadership in vaccination gets highlighted.
• The need for incentive-based programmes for vaccination emerges as significant.

## 1 Introduction

In 2014, the World Health Organization’s Strategic Advisory Group of Experts (SAGE) defined the concept of vaccine hesitancy (reluctance toward vaccination despite availability) [1]. However, vaccine hesitancy is not a recent phenomenon and has existed since the first vaccine was administered about 200 years ago. Reluctance to accept the measles vaccine in parts of Europe, the hepatitis (HepA and HepB) vaccines in U.S., the human papillomavirus (HPV) vaccine in Japan and India, and the polio vaccine in parts of Nigeria and Pakistan, are some examples of vaccine hesitancy from different parts of the world [2], [3], [4], [5]. The development and severity of vaccine hesitancy is individualized and depends on one’s exposure to various social, political, and environmental influences. In 2019, the WHO classified vaccine hesitancy as a top 10 global health threat [6]. Since then, the determining factors of vaccine hesitancy have been explored in several settings.

COVID-19 pandemic, triggered by the rampant spread of the SARS-CoV-2 virus, has sparked public health crisis and constraints on the daily lives of people across the globe. The World Health Organization (WHO) has declared the viral infection as a pandemic on March 11, 2020. In India, the first case of COVID-19 was confirmed on January 27, 2020, and cases spread from one state to other states and the union territories of India. The SARS-CoV-2 virus has caused over six million deaths and close to 600 million reported cases worldwide, as of September 15, 2022, and the death toll in India is over 528,250 [7].

Prevention behaviours for avoiding contracting the disease are well understood, including wearing facial masks, washing hands, physical distancing and avoiding crowds. However, several studies have indicated variations in tendency to follow protective measures according to individual’s socio-demographic factors (e.g., age, gender, educational level, income), trust in several entities (e.g., scientists, government, medical system), perceived threat and risk of the coronavirus disease, and political ideologies [8]. Indian Government has taken urgent measures to expand the country’s vaccine manufacturing capacity and developed an efficient digital system to address and monitor the vaccine uptake and administration [9]. Despite these efforts, in India a perpetual paradox, is grappling with the fear of getting infected and wait anxiously to get the COVID-19 vaccine, and besides, the issue of vaccine hesitancy in spite of availability of vaccination services. Vaccine hesitancy remains a barrier to full population inoculation against highly infectious diseases [10]. For instance, in India, vaccine hesitancy towards influenza pneumococcal, typhoid, hepatitis B and diphtheria/tetanus/pertussis (Tdap) persists owing to the presence of certain misconceptions among adults [11], [12]. Evidence shows a significant proportion of the population remains vaccine hesitant, with almost a third (29 %–39 %) of the population being vaccine hesitant in early 2021 [13]. Additionally in context of COVID-19 pandemic, 7 per cent of adult population of India are hesitant to take the vaccine as of October 2021 [14], considering India’s population the numbers are of significant proportion.

Studies from the early phase of pandemic show health systems in low- and middle-income countries (LMICs) struggle to address COVID-19 [15], vaccine access has become a cause for national and international concern. Most LMICs have been slow to receive and distribute vaccines whereas vaccines are abundantly available in high-income countries, promoting critiques of global vaccine inequality [16], which were exacerbated with the distribution of so-called booster shots in high income countries in autumn of 2021. Additionally, combined with the identified access issues the higher health risks of vaccine hesitancy among the adults is having catastrophic effects. Vaccine hesitancy or delayed vaccination increases the risk of viral diseases such as Pertussis, Measles and Haemophilus influenzae B infections up to six folds [17], [18], [19], [20]. This highlights the need to augment efforts to increase vaccine equity and public trust in vaccination [21].

The emergence of coronavirus variants, concerns around waning immunity with primary vaccine series and evidence from other countries were the primary drivers of initiating booster dose vaccination in India in January 2022 [22]. India's Covid graph inches upwards, not enough people are getting their booster shots [23]. Reports suggest more than 20 % hesitant to receive the booster dose among the population who received the first two doses of vaccine [24]. Considering the total population of India, 20 % forms a significant figure, thus highlighting the need to understand the determinants of vaccine hesitancy in details.

Traditional theories of behaviour propose individuals protect themselves based on cognitive factors such as threat appraisal (Protection Motivation Theory) [25] or perceived susceptibility/severity of an illness (Health Belief Model) [26]. Both the Theory of Planned Behaviour (TPB) and the Theory of Reasoned Action (TRA) posit an individual’s attitude toward a behaviour and perceived social norms surrounding the behaviour contribute to an individual’s intention, or motivation to carry out the behaviour [27], [28]. According to these perspectives, increasing the accuracy of people’s perceived social norms about the threat or severity of an illness ought to elevate the probability of adopting steps to protect themselves against diseases/viruses (e.g., by vaccination uptake).

Furthermore, a number of studies have found a link between vaccine incentives and avoidance of risk behaviours. For example, Verma et al., 202 used the Prospect Theory to explain the association between COVID-19 gain and loss frame messages could be deployed strategically to promote SARS-CoV-2 vaccination depending on how the target audience perceives the relative risks and benefits of vaccines, as well as their self-perceived risk of suffering severe consequences of COVID-19 (e.g., economic incentives of the vaccination to adoption of preventive behaviours such as intention to get vaccinated) [29], [30].

Given the importance of perceived social norms in forming the behaviour pattern with respect to the vaccination and incentive-based intervention as a potential motivator to receive vaccine, the current study attempts to assess a population-representative sample of adults from India in order to identify adult vaccine hesitancy factors. First, the socio-demographic determinants of hesitancy are outlined to detail the profile of vaccine hesitant people. Second, the predictors of vaccine hesitancy are predicted through perceived social norms, including perceived injunctive norms (e.g., community and political ideology, and timely and reliable information), and perceived descriptive norms (beliefs and behaviours, preferences, and vaccine experiences). Third, we bring out the role of incentive-based intervention to improve vaccine acceptance and speed up the process of vaccination in India. Community-based cross-sectional survey data were collected from 11 diverse states. The survey was conducted from July-November 2021, taking observations of 1129 adults, above 18 years old.

The study builds upon existing literature. The World Health Organization’s 5Cs model forms the popular choice to determine the psychological causes of vaccine hesitancy. The model uses five drivers of vaccine hesitancy, namely, complacency, convenience, confidence, calculation, and collective responsibility [31], [32]. Annexure 1 presents the conceptual model of the 5C drivers of vaccine behaviour. The 5Cs’ model has been used to investigate several aspects of vaccine hesitancy, including the role of community allergy/immunology [33]; perceptions of Covid-19 vaccine and identification of a feasible, community-centred solution [34], [35]; and psychological determinants of vaccination behaviour [36], [37].

However, the 5Cs model does not include social factors (such as social networks, community, and political leaders) particularly in adults who have a higher exposure to social and political impacts [38], [39]. Adult behaviour and attitude get affected by the behaviour and attitude of peers [40]. Social norms influence behavioural choices to significantly determine health-related behaviours like physical activity, eating behaviour, and vaccination behaviour [41], [42]. For example, vaccine opinions of neighbours forms a powerful predictors of decisions for vaccine acceptance or refusal [43]. Further, medical and governmental factors such as distrust, uncertainty about content of vaccine and fear of side effects directly impact social behaviour, and indirectly impact vaccine hesitancy [44]. Perceived social norms, thus, may play a key role in COVID-19 vaccination uptake [45].

Social norms fall within two broad classes namely, perceived descriptive norms and perceived injunctive norms [46]. Perceived descriptive norms refer to people’s beliefs about commonly held behaviours practices in a society (e.g., vaccines prevent disease), and depending on the context, may hinder or inspire one to get a vaccine [47]. For instance, people’s distrust in political leaders could impact the willingness to take up vaccine program and further, causes antivaccination behaviours in a country [48], [49]. Injunctive norms refers to the belief about what other people expect them to do and directs then on what they ought to do [41], [42]. In other word, injunctive social norms, with respect to vaccination, take the form of encouraging behaviour perceived as ‘normal’ or ‘right thing to do’ and socially responsible [50]. Therefore, other than the conventional 5Cs’ model, understanding social norms and behaviours becomes imperative as well.

The study contributes to literature on vaccine hesitancy in two ways. One, the study goes beyond the popular 5Cs model to explore the role of social norms in persistence of vaccine hesitancy. Perceived descriptive norms and perceived injunctive norms are explored in the Indian adult population. Two, the study explores the role of political and community leadership in influencing vaccine hesitancy/uptake. The impact of political leadership has been discussed recently in popular literature and the significant impact of the leadership has been highlighted in developing trust/mistrust among individuals. The article explores the same through the academic lens. The results may also provide more general insights into the importance of the incentive-based interventions as among the successful measures for increasing vaccine uptake in context of Indian adults.

The paper contains five sections. Section 2 presents the method used for analysis, including the theoretical foundation and data analysis. Section 3 present the results of the analysis, followed by a discussion on the key findings in Section 4. Section 5 presents the conclusion and implications derived from the study.

## 2 Methods

### 2.1 Study design

Modern India stands as one of the most diverse countries in the world, with 29 states, each with their own unique languages, traditions, religions, and beliefs. Vaccine hesitancy presents in diverse communities in India–including across socio-economic status, urban and rural settings, and among different religious communities. Several studies stated vaccine hesitancy had historically been observed among lower income communities, particularly in the context of polio campaigns. Evidence shows while the majority of Indians would accept the vaccine, given the large population of India, even a small proportion of hesitant individuals would translate to millions of unvaccinated individuals [51]. The recent studies demonstrate the states such as Tamil Nadu, Punjab, Nagaland, Meghalaya, Mizoram, Manipur, Jharkhand, etc. have high vaccine hesitancy and relatively low vaccination coverage [52].

### 2.2 Data collection

The study is a cross-sectional quantitative study using a population-representative sample. Participants were adults with age 18 years and older, chosen by cluster sampling method from those who had benefited from the Government free food scheme in July-November 2021, across 11 states of India where the food distribution centres are located. Participants were provided free food credit for sharing their number and time for phone call interview. The data was collected during July-November 2021, through a telephonic interview. The free food beneficiaries were contacted maximum three times by telephone (mobile phone) for a conversation. 1131 adults from across different states in India responded to the phone call. The process of questions and answers were recorded. Two participants were excluded for not completing some vaccine behaviour questions. We had about 22 % success rate.

#### 2.2.1 Ethnic and data approvals

Institutional Review Board (IRB) approval was taken from the Akshaya Patra Foundation. The approval allows for use of the data for academic analysis and reporting. The ‘data use approval letter’ is attached at the end of the manuscript.

### 2.3 Analytical strategy and survey measures

Our analyses investigate the association between baseline indicator of COVID-19 vaccine acceptance during pandemic and factors determinants of vaccination. The survey measures and a supplem*****entary full list of the applied survey instruments are presented below.

The structured questionnaire includes three sections. The first section comprises single-choice questions to determine the sociodemographic characteristics of participants. The second section comprises multiple choice questions to assess the acceptance of COVID-19 vaccination and social norm impacts, including questions on trust/distrust of vaccines, hospitals, and credible vaccine information. The third section comprises five multiple choice propositions to probe incentives influences.

#### 2.3.1 Demographic characteristics

Participants were asked about their gender identity, age, education, employment status, family strength, place of residence, and household income per month. Responses were categorized into two, man = 1 was compared to individuals who identified as women = 2. Participants were asked the education level and nature of job as well.

#### 2.3.2 Vaccine social norms

Perceived descriptive norms were assessed with the questions related to (a) participant’s trust or mistrust, “Do you feel safe/protected after being vaccinate?”, “Do you feel we can rely on vaccines to stop serious infectious diseases?”, “Do you think vaccines can cause unseen physical problems?”, and “Do you think vaccines can weaken the body in long-term?”, (b) participant’s personal vaccine experiences with the questions, “Have you ever had bad experience with vaccination” and “Do you know someone with a bad vaccine experience?”, (c) participant’s vaccination preferences with the questions, “Do you prefer natural immunity rather than vaccination or do you feel vaccination is necessary if you use natural medicines and remedies?”, and (d) participants credible vaccine information with the questions “Do you get information about various vaccines from time to time?”, “Do you feel hospital/health-center staff is competent on vaccinations?”, “Do you feel vaccine given by your doctor or local health unit is safe?”, “Do you feel afraid to get the COVID-19 infection?”, and “Have you had reliable information about COVID-19 vaccination?”.

Perceived injunctive norms were assessed with the questions, related to the leadership influences in vaccination. These questions were “Have your community leaders encouraged COVID vaccination?”, “Have your political leaders encouraged COVID vaccination?”, “Do you think community leader motivates you to vaccination?”, and “Do you think community leader motivates you to vaccination?”. Regarding the perceived descriptive and injunctive norms, the response format was a five-point Likert scale from perceived severity of COVID-19. Response options to all the questions were coded as No = 1, Mostly no = 2, Cannot say = 3, Mostly yes = 4, Yes = 5.

#### 2.3.3 Incentive-based intervention

The questions regarding incentives were on various cash incentives, cash vouchers, provision of food for the family or children, benefit like cloths and books, assured cash prizes and gifts, and school for children.

### 2.4 Respondent characteristics

The final sample was 1129 Indian adults from 11 states, namely, Uttar Pradesh (n = 79), Delhi (n = 174), Uttarakhand (n = 2), and Haryana (n = 8) located in north-central India, Telangana (n = 368), Tamil Nadu (n = 4) located in south-central, Andhra Pradesh (n = 2) located on south-eastern part of the Indian subcontinent, Karnataka (n = 451), and Maharashtra (n = 2) located on the country's western coast, and West Bengal (n = 7), and Bihar (n = 8) is located in the eastern part of India. At the time of administration, the COVID-19 vaccine had been made available to some adults with certain groups (e.g., at risk adults and healthcare workers) receiving priority and the roll-out across the India was ongoing. In each of the states where data were collected, approximately 81 % of adults in the general population were estimated to have received a vaccine for COVID-19 when the survey started [53]. Despite the India rolled out the world’s largest vaccination drive to vaccinate around 300 million priority groups against the coronavirus disease (COVID-19) (as per January 2021) [54], the phenomenon of COVID-19 vaccine hesitancy appeared more pronounced among tribal populations in some states including Maharashtra, Gujarat, Odisha, Chhattisgarh, Bihar, Rajasthan, Jharkhand, Karnataka, Andhra Pradesh, and West Bengal in India (as per February 2022) [55].

While we find disincentives from the states like threatening public announcements and holding back of food rations [56], [57], [58], several states have given direct incentives through the government and private agencies. In the South state of Tamil Nadu, a Chennai-based Foundation provides lucky draws with prizes such as gold coins, washing machines, blenders and bikes for people taking the vaccine [59]. The Sheohar district administration in Bihar adopts the same model to overcome vaccine hesitancy [60]. The goldsmith community in the state of Gujarat has provided freebies like gold nosepin and hand blender to encourage people for vaccination [61]. The Jan Swasthya Abhiyan of the state of Rajasthan proposes fully vaccinated families be given 150 days of MGNREGA work instead of the regular 100 days and 7 kg of grain per person per month instead of 5 kg. Further, pregnant and lactating mothers receive one free hot cooked meal after two shots of vaccination [62]. The Chandrapur Municipal Corporation in the state of Maharashtra has announced a vaccination bumper lucky draw, with lucrative prizes ranging from LED TVs, refrigerators and washing machines [63]. These incentives contribute to encouraging more people to get inoculated against COVID-19. However, to understand the main causes of the vaccine hesitancy issues along with these incentive-based interventions, remains a gap.

The present study explores the relationship between influential factors and the likelihood of vaccine acceptance among Indian adults across different locations with higher prevalence of COVID-19 vaccine hesitancy. See Table 1 for differences among states.

**Table 1:** Descriptive statistics.

Variable	Total (N = 1129)	
	COVID-19 vaccinated (n = 915)	COVID-19 non-vaccinated (n = 214)
**Gender**		
Male	63.1	68.0
Female	36.9	32.0
**Education**		
Primary	27.2	18.9
Secondary	39.1	46.5
High School	16.6	16.5
Graduation	16.6	15.2
Post-Graduation and above	0.6	3.0
**Employment status**		
Student or Unemployed	21.5	15.7
Labour work	22.0	17.8
Business or Self employed	36.0	53.1
Healthcare provider	20.6	13.0
Govt Employee	0.0	0.2
**State**		
Uttar Pradesh	18.2 %	4.7 %
Telangana	22.7 %	35.7 %
Delhi	19.2 %	15.0 %
Karnataka	36.9 %	41.7 %
West Bengal	1.0 %	0.6 %
Uttarakhand	0.0 %	0.2 %
Haryana	0.0 %	0.9 %
Bihar	1.0 %	0.7 %
Maharashtra	0.0 %	0.2 %
Tamil Nadu	1.0 %	0.2 %
Andhra Pradesh	0.0 %	0.2 %

In general, participants were, on average, 34 years old (SD = 10). Across the sample, 67.1 % were men, and 32.9 % were women. 20.7 % participants had completed the primary level education, 16.5 % were lower than secondary level, 28.4 % had completed the secondary level, 16.5 % had completed senior secondary, and 18 % had completed graduation and above. Of the sample 16.8 % identified as without job/unemployed, 30 % as labour (construction, and farm), 14.4 % as business or self-employed, 0.2 % as healthcare provider, and 19.9 % as government employee.

Table 1 illustrates the COVID-19 vaccinated, and non-vaccinated participant characteristics. Two interesting results differentiate the two groups. The vaccinated adults are less educated than the non-vaccinated adults. A higher number of vaccinated adults have only primary education and a higher number of postgraduates and above are found in the non-vaccinated group. Furthermore, surprisingly, a higher proportion of business or self-employed (53.1 %) group are hesitant towards the COVID-19 vaccine. These results are discussed in the further sections.

### 2.5 Outcome measures

The study uses COVID-19 vaccine acceptance as the dependent variable. Participants were coded 0 = No if they had not received the vaccine and did not intend to get the vaccine and coded 1 = Yes if they have received at least the first dose.

### 2.6 Predictor variables

The independent variables had four sections, namely, (a) socio-demographic characteristics, (b) perceived descriptive norms (how group belief influences an individual) such as vaccine behaviour, beliefs, preferences, and experiences, (c) perceived injunctive norms (what is acceptable behaviour) such as community and political leadership, (encouragement and motivating role), timely and reliable vaccine information, and (d) significance of incentives such as gifts, rewards, and monetary/financial incentives.

### 2.7 Statistical analysis

T-test, Exploratory Factor analysis, and Binary Logistic Regression were used. T-test assesses the significant variations between the vaccinated and non-vaccinated participants. Since the set of independent variables have multiple dimensions, certain variables may explain similar information leading to multicollinearity issues in the analysis. To address this, we employ Exploratory Factor Analysis (EFA) to uncover the underlying dimension explaining the variation among variables and reduce the dimension of the analyses to the set of independent factors explaining maximum variance. Principal Component Method with varimax rotation extracted the factors with eigenvalue greater than 1.

Table 2 presents the factor loadings on each factor. Together these factors explain 67.1 % cumulative variance. Seven components got extracted, namely, beliefs on safety of vaccine, impact on physical health, experience with vaccine, confidence in medical staff, information about vaccine, leadership ideology influence, and incentives to vaccine.

**Table 2:** Factor Analysis results: The component matrix for principal component analysis (seven components have been extracted).

Factors	Variables of interest	Components						
		1	2	3	4	5	6	7
Belief safety of vaccines **1**	Believes vaccines are safe	0.035	0.034	0.206	−0.255	0.594	−0.062	0.109
	Vaccines can prevent serious illness	0.042	−0.013	−0.005	0.181	0.773	−0.028	−0.063
Impact on physical health **2**	Vaccines can cause unseen physical problems	0.050	−0.001	−0.201	0.199	−0.062	0.461	−0.054
	Vaccines can weaken the body in the long term	−0.058	−0.027	0.281	−0.281	−0.030	0.672	0.015
Experience with vaccine **3**	Had bad experiences with vaccination	−0.034	0.018	−0.066	0.759	0.016	0.150	0.093
	Knows someone with a bad vaccine experience	−0.010	−0.045	0.006	0.775	−0.003	0.043	−0.024
Confidence medical staffs **4**	Hospital/health centre staff is competent	0.012	−0.070	0.722	−0.149	0.204	0.026	0.283
	Vaccines given by doctors are safe	0.052	−0.010	0.784	0.110	0.180	−0.022	0.021
	Afraid of COVID infection	0.005	−0.043	−0.464	0.071	0.182	0.043	0.173
Information about vaccine **5**	Had reliable information on vaccination	0.014	0.033	−0.181	0.229	−0.108	−0.106	0.786
	Gets timely information	0.033	0.038	0.282	−0.147	0.139	−0.014	0.582
Leadership influence **6**	Community leaders encouraged everyone to vaccinate	0.036	0.835	−0.086	−0.043	−0.068	0.095	0.187
	Political leaders encouraged everyone to vaccinate	0.005	0.842	−0.082	−0.056	−0.077	0.138	0.161
	Got motivated due to community leader	0.161	0.826	0.072	0.040	0.065	−0.101	−0.119
	Got motivated due to political leader	0.138	0.825	0.085	0.022	0.090	−0.072	−0.153
Incentives to vaccines **7**	One day salary	0.808	0.073	0.161	−0.034	−0.102	−0.091	−0.054
	Three days salary	0.807	0.058	0.239	−0.052	−0.103	−0.099	−0.095
	One month ration	0.768	0.106	0.199	−0.057	−0.189	−0.151	−0.049
	Free food for children	0.825	0.088	0.140	−0.033	−0.105	−0.099	0.001
	Kitchen gifts or silk dress	0.867	0.063	−0.169	0.045	0.114	0.131	0.066
	Automobile gifts, household gifts or silk dress	0.869	0.057	−0.175	0.039	0.123	0.145	0.065
	300 Rupees for each vaccination	0.802	0.025	−0.160	0.015	0.248	0.153	0.079
	500 Rupees for each vaccination	0.811	0.012	−0.165	0.028	0.242	0.115	0.062

Subsequently, we employ Binary Logistic Regression model to probe the influence of independent variables including variables extracted from factor analysis (see Table 2) as well as demographic variables (such as age, gender, education, family strength, and household income per month) on COVID-19 vaccine acceptance. We used the Statistical Package for Social Sciences (SPSS) software program version 25.0 for all analyses. All statistical tests descried were two-sided, and analysis items with common significance levels, P-value < 0.01 (1 chance in 100), P-value < 0.05 (1 chance in 20), P-value < 0.1 (1 chance in 10).

The 95 % confidence interval (CI) is used to estimate the precision of the odds ratio (OR). A large CI indicates a low level of precision of the OR, whereas a small CI indicates a higher precision of the OR. The OR is used to determine whether a particular exposure is a risk factor for a particular outcome (COVID-19 vaccine uptake), and to compare the magnitude of various risk factors for the outcome. The odds ratio is defined as follows:

1. OR = 1 Exposure does not affect the odds of the outcome.
2. OR > 1 Exposure associated with higher odds of the outcome.
3. OR < 1 Exposure associated with lower odds of the outcome.

## 3 Results

### 3.1 Difference between vaccinated and vaccine hesitant participants

The comparison of the means of variables across the two groups of vaccinated and vaccine hesitant/non-vaccinated (Table 3) brought our four significant differences between the vaccinated and the non-vaccinated.

(a) Vaccinated group have a significantly lower monthly income (M = 15669.93) than the non-vaccinated people (M = 16476.74). Individuals from higher income households are seen as less likely to take vaccination as compared to low-income households.
(b) Perceived injunctive norms in terms of the political and community leaders fail to make a positive impact on vaccination. The vaccinated group have a lower mean value (M = 2.88) than the non-vaccinated group (M = 3.04).
(c) Influence of perceived descriptive norms were higher among the vaccinated group on vaccine safety (M = 4.73) as compared to the non-vaccinated (M = 4.17). Similarly, the non-vaccinated have a higher mean value (M = 2.75) than the vaccinated group (M = 2.67). Participants who perceived the hospital/health centre staff as competent are significantly more likely to fall under the vaccinated group (M = 4.31) than the non-vaccinated group (M = 3.86).
(d) Monetary/financial incentives might have a positive impact on vaccination. The vaccinated group show higher mean value (M = 3.18) as compared to the non-vaccinated group (M = 2.90).

**Table 3:** Levene's Test for Equality of Variances.

		Vaccine for COVID	Mean	F.	sig.	t
**Demographic characteristics**	Household income per month	Non-vaccinated	16476.74	3.99*	0.05	0.43
		Vaccinated	15669.93			0.30
**Perceived descriptive norms**						
Vaccine behaviours and beliefs	Believes vaccines are safe	Non-vaccinated	4.17	164.92***	<0.001	−8.18
		Vaccinated	4.73			−6.10
	Vaccines can weaken the body in the long term	Non-vaccinated	2.75	17.52***	<0.001	0.64
		Vaccinated	2.67			0.68
	Hospital/Health centre staff is competent	Non-vaccinated	3.86	25.51***	<0.001	−5.43
		Vaccinated	4.31			−4.97
Vaccine experience	Had bad experiences with vaccination	Non-vaccinated	2.05	21.72***	<0.001	−2.25
		Vaccinated	2.30			−2.51
**Perceived injunctive norms**						
Community and political leadership	Political leaders encouraged everyone to vaccinate	Non-vaccinated	3.04	5.37**	0.02	1.33
		Vaccinated	2.88			1.37
	Got motivated due to community leader	Non-vaccinated	3.23	4.12**	0.04	1.01
		Vaccinated	3.13			0.97
	Got motivated due to political leader	Non-vaccinated	3.22	3.57**	0.06	1.00
		Vaccinated	3.12			0.96
**Vaccine incentives**						
Monetary/financial incentives	300 Rupees for each vaccination	Non-vaccinated	2.90	3.81*	0.05	−2.81
		Vaccinated	3.18			−2.63
	One month ration	Non-vaccinated	3.51	4.21**	0.04	0.38
Rewards/gifts incentives		Vaccinated	3.47			0.37
	Free food for children	Non-vaccinated	3.38	10.17***	<0.001	0.00
		Vaccinated	3.38			0.00

Non-significant variables include Family strength, Education, Statements: Vaccines can prevent serious illness, Vaccines can cause unseen physical problems, Vaccines given by doctors are safe, Afraid of COVID infection, Preferences for natural immunity, Know someone with bad vaccine experience, get timely information on vaccination, get reliable information on vaccination, One/Three day salary, Automobile gifts, household gifts like silk dress, Schools reopening for children, and Back to normal life.

Significance determined at ***p < 0.01, **p < 0.05, *p < 0.1.

### 3.2 The socio-demographic determinants of vaccine acceptance

Table 4 describes the logarithmic association between vaccination and the contributing factors. Education emerges as significant (p-value < 0.02, 95 % CI 1.06–1.83), confirming the literature evidence on education promoting vaccination and optimizing efforts to address hesitancy among individuals [64].

**Table 4:** Regression analysis.

Factors	B	Sig.	Exp (B)	95 % C.I. for EXP(B)	
				Lower	Upper
Gender (Male - Female a)	0.54	0.16	1.71	0.81	3.59
Age	0.02	0.22	1.02	0.99	1.06
Household Income (per month)	0.00	0.19	1.00	1.00	1.00
Family strength	−0.05	0.58	0.96	0.81	1.12
Education	0.23*	0.05	1.25	1.01	1.56
Vaccine incentives	0.33**	0.02	1.39	1.06	1.83
Community and political ideology	−0.28*	0.06	0.76	0.57	1.01
Confidence medical staff	0.10	0.50	1.10	0.83	1.47
Experience with vaccine	0.10	0.52	1.10	0.83	1.46
Belief safety of vaccines	0.52***	<0.001	1.69	1.29	2.21
Impact on physical health	−0.03	0.84	0.97	0.73	1.29
Information about vaccine	0.43***	<0.001	1.53	1.14	2.05

a Represents reference level; CI indicates confidence interval; Significance determined at ***p < 0.01, **p < 0.05, *p < 0.1.

Interestingly, income does not play a role in vaccine willingness. However, the comparison of income mean between non-vaccinated and vaccinated groups (Table 3) shows the probability of higher income households accepting COVID-19 vaccine being lower as compared to low-income households.

### 3.3 The role of perceived social norms in triggering vaccination hesitancy

The perceived injunctive norms in the community and political leadership contribute negatively to the likelihood of vaccine acceptance (p-value < 0.06, 95 % CI, 0.57–1.01). Literature infers political leadership positively influencing vaccination acceptance among the adult population [49], [65]. Our results demonstrate otherwise.

The injunctive norm of timely availability of reliable vaccine information (p-value < 0.001, 95 % CI, 1.14–2.05) gets confirmed through the results. Previous literature finds accurate information for vaccination (e.g., information on health determinants, health systems performance, health status of vaccinated individuals, and accurate immunization coverage) leading to progress toward vaccination coverage goals [66], [67], [68]. Our results also confirm the same.

The perceived descriptive social norms of belief in safety of vaccines increases the likelihood of vaccination uptake (p-value < 0.001, 95 % CI, 1.29–2.21), thereby highlighting the tempering effect of safety beliefs on the initial intentions [69].

### 3.4 The role of incentive-based intervention in likelihood of vaccination

The significant association between different types of vaccine incentives (such as monetary/financial, rewards/gifts, motivational incentives) and increasing likelihood of vaccination acceptance (p-value < 0.05, 95 % CI, 1.01–1.80) among participants comes out clearly. The result confirm recent literature emphasizing the effectiveness of nudge-based interventions include using incentives to increase vaccine confidence and encourage vaccine uptake [70].

## 4 Discussion

The current study sought to understand the determinants of vaccine acceptance among Indian adults. Adding to earlier research, the importance of perceived social norms for vaccination were explored. Broadly, three interesting factors stand out as significant contributor to the acceptance of the COVID-19 vaccine. First, the positive effect of education, negative impact of income, and negative impact of self-employment occupation. Second, the role of perceived social norms of community and political leadership and appropriate information. Lastly, the role of monetary/financial incentives as a predictor of vaccine related decision making among Indian adults.

### 4.1 The socio-demographic determinants of vaccine acceptance

Occupational differences were seen in the *t*-test and descriptive statistics. A higher proportion of business or self-employed (53.1 %) were hesitant towards the vaccine. Literature on vaccine hesitancy by occupation reports distrust of the government among the most pronounced reasons of vaccine hesitancy in self-employed, and comparatively more concerned about vaccine safety [71]. Research found workplace conditions like encouragement from colleagues and talks by health workers as significantly impacting vaccine uptake (indirectly highlighting the importance of social norms!) and the authors recommended vaccine messaging in employers’ safety practices. Self-employed possibly miss out on this important workplace dynamics [67], [68].

Results confirm literature evidence on higher level of education being a protective factor against vaccine refusal. Greater distrust is seen for medical professionals amongst communities with less formal education [72]. Lower education level also creates a hurdle in possessing correct information regarding vaccination [48].

### 4.2 The role of perceived social norms in triggering vaccination hesitancy

Our findings show strong role of the perceived injunctive norms on vaccine hesitancy/vaccine uptake. In contrast with earlier literature, we find negative influence of leaders in increasing the use of health services. For instance, in South Africa, the social/community leaders have had a significant impact on HIV and AIDS prevention [73]. The social and political leaders played an integral role in scaleup of polio campaigns in northern Nigeria [74]. Our results depict community and political leaders negatively impacting the likelihood of receiving vaccine among Indian adults.

Popular literature however highlights a contention to the above. Political and community leaders can also sway the public away from receiving vaccination by conveying false information on the virus, the vaccine and/or the health care system [75], [76]. For instance, in the US, republican political and thought leaders have downplayed the virus and failed to encourage vaccination. The ex-President of the USA, Mr Donald Trump, promoted claims on the virus being combatted by injecting or drinking disinfectant or bleach [75], [76]. Republican leaning counties have failed to implement safety measures, failed to get a high proportion of residents vaccinated, and as a consequence suffered higher COVID-19 death rates [77]. We may be witnessing the same in India.

The adverse impact of leadership ideology could be attributed to the novelty of the virus as well. As the information regarding the novel coronavirus remains limited, the likelihood of the leaders themselves not being entirely certain about the effective and safety of vaccines remains high. The significant importance of timely and reliable information found in the results support this. Hence, conveying sufficient and transparent information (e.g., quality information on how vaccines are developed and tested, and how vaccines work) by professionals and trusted sources stands out as critical for improved vaccine coverage. This may counter the negative influence of leadership as well.

### 4.3 The role of incentive-based intervention in likelihood of vaccination

The effectiveness of nudge-based interventions includes using incentives to as a vital leverage to enhance the positive attitudes towards vaccination. The study brings out specific incentives for the critical coverage levels necessary to control the epidemic. Significant financial incentives include monetary incentives (e.g., cash payments) and non-monetary incentives (e.g., meal vouchers, rewards/gifts) as significant to address vaccine/immunization uptake.

However, these programs can have supersizing effects [78]. Studies demonstrate people reacting differently depending on who provides the incentives and the corresponding levels of trust in receiving the promised incentives [79]. Hence, the incentive-based vaccination programs should be recommended by trusted community, social, political, and healthcare leaders and carefully evaluated before they are implemented.

### 4.4 Policy implications and research implications

Contrary to existing literature, we found limited role of political and community leaders as a driver of behavioural health in terms of vaccine hesitancy. However, these intentions can be modified by trusted health communicators. Our opportunistic exploratory study suggests political speaker’s endorsement of the COVID-19 vaccine may reduce the uptake of vaccines. As per the World Health Organization (WHO), healthcare professionals and public health authorities have a central role in discussing vaccination against COVID-19 with their patients [80]. Further, the health expert’s factually accurate message contributes to the individual confidence in national safety monitoring systems, controlling the spread of the disease and supporting people in making the critical choice to be vaccinated. To counter vaccine hesitancy, future research and practice can explore communication strategies for specific segments of the population.

Consistent with other studies, we found incentive-based vaccination programs as most effective ways to limit and overcome the COVID-19 pandemic. Hence, the well-designed and well-publicized incentive programs can support efforts to achieve vaccine coverage in India. Similarly, studies found in the United States (U.S.), North Carolina [81], and in Europe, Sweden [79], the guaranteed payment incentives for adult vaccination bolstered vaccine uptake. However, the effectiveness of vaccine incentives may also depend on cultural norms and beliefs and may thereby differ across countries [82].

### 4.5 Strengths and limitations

To our knowledge, our study forms the first cross-sectional study examining perceived social norms factors associated with vaccine hesitancy and parallelly bringing out the potential protective role of community and political leadership as well as incentive-based intervention among population representative sample of adults across diverse states in India. We collected data from a large sample in eleven states assessing vaccine acceptance, and hesitation in various populations from different contexts, cultures, and backgrounds. The study contains sampled population were from most reported hesitant states such as Karnataka, Telangana, and Delhi [52] with disseminating information on the safety and efficacy of COVID-19 vaccines. Moreover, a higher proportion of the sampled population were from business or self-employed status includes high rates of belonging to informal labour which has experienced with dramatical descending changes in economic status due to the pandemic restriction. Hence, the study provides the appropriate information of the population representative sample of vaccine hesitant adults.

Despite these strengths, the present study includes two limitations. First, response biases could be one of the critical limitations of the study. Another significant limitation is the representativeness of the sample population. A higher proportion of the sampled population were educated. Since, hesitancy was slightly lower among educated population, overrepresentation of these groups could lead to underestimation of vaccine hesitancy. Lastly, the percentage of older people were higher than younger adults the COVID-19 vaccine hesitancy data in our study.

## 5 Conclusion

In the current study we go beyond the most common framework of 5Cs model by studying the potentially crucial role of perceived social norms in impacting the vaccination acceptance among adults. The findings demonstrate some confirmatory and some contradictory results to literature. For instance, political and community leaders may not be able to leverage their role in positively influencing and educating people about the criticality of vaccines. However, timely and transparent information can plan a strong role to counter this effect and propagate positive attitude towards vaccines to achieve the vaccination goal in India.

The most promising evidence exists for nudges (altering the environmental context in which a decision needs to be made) that offers incentives to improve vaccine uptake [70]. Evidence shows guaranteed small monetary/financial incentives as a powerful motivator tool to offset costs related to vaccination (e.g., costs of transportation, childcare, and lost wages) particularly in population with higher economic vulnerability [81]. Our results suggest the same as well. COVID-19 vaccine incentives can influence the likelihood of vaccine acceptance.

The findings emphasize on garnering the attention of local and public health services, health protection practitioners and national media to induce vaccine literacy among adults by educating them about the importance of vaccination for the global health. Vaccine literacy contributes to understand the basic health information and services to make appropriate health decisions and further, encourage vaccines uptake. The public health interventions such as communication strategies, normative messaging, and use of telemedicine as a practice to provide health care, which has been adopted in mass vaccinations during previous disease outbreaks [83] are crucial to circulate the correct information, spread awareness, and improve perceptions of vaccine safety and effectiveness.

## Declaration of Competing Interest

The authors declare that they have no known competing financial interests or personal relationships that could have appeared to influence the work reported in this paper.

## Figures and Tables

**Figure:**
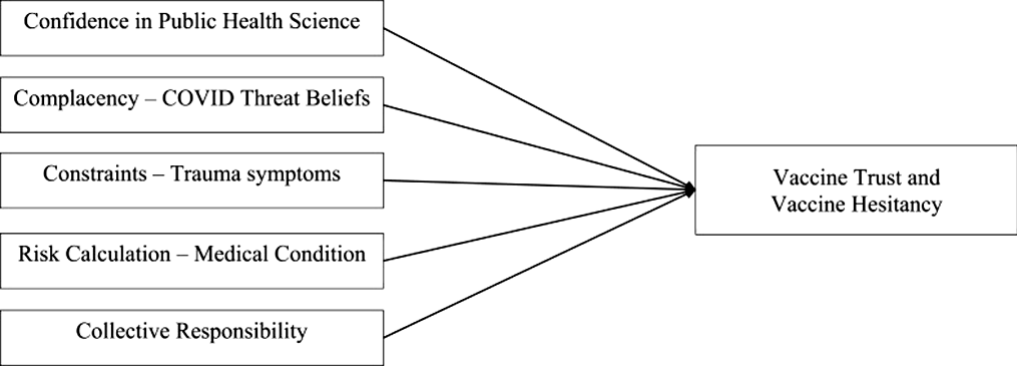


## Data Availability

The authors do not have permission to share data.

## Annexure 1 Conceptual model of 5C drivers of vaccine behaviour

Source: [84].

Note. The 5C model of the drivers of vaccine behaviour provides five main individual person–level determinants for vaccine hesitancy: confidence, complacency, constraints, risk calculation, and collective responsibility.
